# 

**Published:** 1878-03-20

**Authors:** 


ORIGINAL AND SELECTED ARTICLES.

Treatment of Hemorrhoids; by L. Q Lincecum, of Texas ....65
The Danger of Using Chloral and Opiates Alternately; by A. R. Kilpatrick, M.D ....68
Extensive Comminuted Fracture of the Leg—Treated Without Splints; by Z. B. Herndon, M.D., of Virginia ....68
Duty of the Doctor to the Dying; by E. L. Smith, M.D., of Texas ....69
Instrumental Labor; by G. McDonald, M.D. ..70
Essay on Pneumonia by Dr. C. H. Hughes ....72
Thoracentesis; by Franklin Staples, M.D ....75
Case of Successful Transfusion Resorted to for Severe Hemorrhage after Delivery; by Drs. Cory and Cameron ....77
Chloral Hydrate; by J. A. Larrabee, M.D ....78
Gurjun Balsam in Gonorrhœa ....79

ABSTRACTS AND GLEANINGS.

Cases of Varicose Veins, 80; Bacteria, and What Will Kill Them, 82; Acute Catarrh of the Ear Ca sed by Snuffing Water up the Nostrils, 82; Raising the Arm in Epistaxis, 82; Chloroform Hallucination, 83; Action of Pilocarpin on the Eye, 83; The Pancreas in Diabetes, 83; Horse Flesh, 84; Influence of Iron Mixed with Food on the Blood, 84; Amputation by Ligature, 35; The Ice-water Treatment of Croup, 86; How to Introduce the Hypodermic Needle, 86; The Blood in Diphtheria, 86.

SOCIETY REPORTS.

The Atlanta Medico-Chirurgical Association; Reported by Dr. Word, 87.

PRACTICAL NOTES AND FORMULÆ.

Pneumonia Aborted by Veratrum Viride, 90; Cinchonia, 90; Constipation, 91; Evaporating Lotion, 91; Hydrocele, 91; Excellent Eye Water, 91; Worm Prescription, 91;

SCIENTIFIC ITEMS.

Sun-spots and Terrestrial Magnetism, 92; The Force of Language, 92; Telephonic Ausculation, 92.

EDITORIAL AND MISCELLANEOUS.

Sample Copies, 93; Stick to Principle, 93; "Allopath" vs. Any Other "Path," 93; Obituary, 94; Book Notices, 94; Counter Statement of Wm. R. Warner & Co., 96.

